# Heptane-1,7-diaminium sulfate mono­hydrate

**DOI:** 10.1107/S1600536811030030

**Published:** 2011-07-30

**Authors:** Charmaine Arderne

**Affiliations:** a Department of Chemistry, University of Johannesburg, PO Box 524, Auckland Park, Johannesburg 2006, South Africa

## Abstract

The crystal structure of the title compound, C_7_H_20_N_2_^2+^·SO_4_^2−^·H_2_O, is presented, with particular focus on the packing arrangement in the crystal structure and selected hydrogen-bonding inter­actions that the compound forms. The crystal structure exhibits parallel stacking of the diammonium dication in its packing arrangement, together with inorganic–organic layering that is typical of these *n*-alkyl­diammonium salts. An intricate three-dimensional hydrogen-bonding network exists in the crystal structure where the hydrogen bonds link the cation and anion layers together through the sulfate anions and the water mol­ecules.

## Related literature

For related structural studies of *n*-alkyl-diammonium sulfate salts, see: van Blerk & Kruger (2008 ▶). For related literature other *n*-alkyldiammonium salts, see: Allen (2002 ▶).
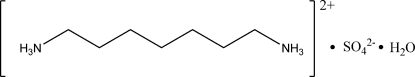


## Experimental

### 

#### Crystal data

C_7_H_20_N_2_^2+^·SO_4_^2−^·H_2_O
*M* *_r_* = 246.33
Monoclinic, 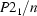

*a* = 5.7527 (1) Å
*b* = 22.3459 (4) Å
*c* = 10.0908 (2) Å
β = 95.180 (1)°
*V* = 1291.87 (4) Å^3^
*Z* = 4
Mo *K*α radiation
μ = 0.26 mm^−1^
*T* = 295 K
0.48 × 0.44 × 0.14 mm

#### Data collection

Bruker SMART CCD diffractometer
Absorption correction: multi-scan (*SADABS*; Bruker, 2008 ▶) *T*_min_ = 0.887, *T*_max_ = 0.965
33427 measured reflections
3220 independent reflections
2626 reflections with *I* > 2σ(*I*)
*R*_int_ = 0.029

#### Refinement

*R*[*F* ^2^ > 2σ(*F* ^2^)] = 0.037
*wR*(*F* ^2^) = 0.106
*S* = 1.03
3220 reflections
138 parameters
H-atom parameters constrained
Δρ_max_ = 0.36 e Å^−3^
Δρ_min_ = −0.34 e Å^−3^

### 

Data collection: *SMART-NT* (Bruker, 1999 ▶); cell refinement: *SAINT* (Bruker, 2008 ▶); data reduction: *SAINT*; program(s) used to solve structure: *SHELXS97* (Sheldrick, 2008 ▶); program(s) used to refine structure: *SHELXL97* (Sheldrick, 2008 ▶); molecular graphics: *OLEX2* (Dolomanov *et al.*, 2009 ▶) and *Mercury* (Macrae *et al.*, 2008 ▶); software used to prepare material for publication: *publCIF* (Westrip, 2010 ▶) and *PLATON* (Spek, 2009 ▶).

## Supplementary Material

Crystal structure: contains datablock(s) I, global. DOI: 10.1107/S1600536811030030/zk2015sup1.cif

Structure factors: contains datablock(s) I. DOI: 10.1107/S1600536811030030/zk2015Isup2.hkl

Supplementary material file. DOI: 10.1107/S1600536811030030/zk2015Isup3.mol

Supplementary material file. DOI: 10.1107/S1600536811030030/zk2015Isup4.cml

Additional supplementary materials:  crystallographic information; 3D view; checkCIF report

## Figures and Tables

**Table 1:** Hydrogen-bond geometry (Å, °)

*D*—H⋯*A*	*D*—H	H⋯*A*	*D*⋯*A*	*D*—H⋯*A*
N1—H1*C*⋯O3^i^	0.89	1.97	2.8617 (17)	176
N1—H1*D*⋯O2^ii^	0.89	1.94	2.8250 (15)	177
N1—H1*E*⋯O4^iii^	0.89	1.92	2.8106 (17)	174
N2—H2*C*⋯O5^iv^	0.89	1.89	2.7643 (17)	168
N2—H2*D*⋯O2^v^	0.89	1.91	2.7859 (15)	166
N2—H2*E*⋯O3	0.89	2.01	2.7770 (16)	144
O1—H1*W*⋯O4^iv^	0.90	1.98	2.880 (2)	180
O1—H2*W*⋯O3	0.90	2.07	2.971 (2)	180

Symmetry codes: (i) 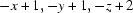; (ii) 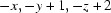; (iii) 
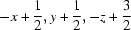
; (iv) 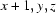; (v) 
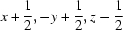
.

**Fig. 1.**
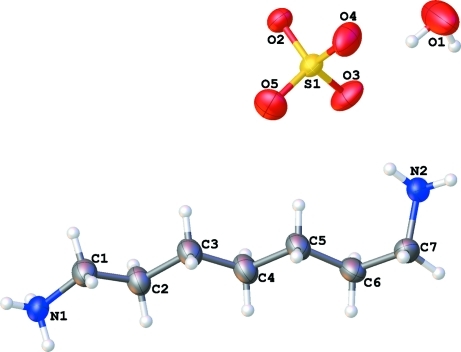
Molecular structure of the title compound, with atomic numbering scheme and displacement ellipsoids drawn at the 50% probability level.

**Fig. 2.**
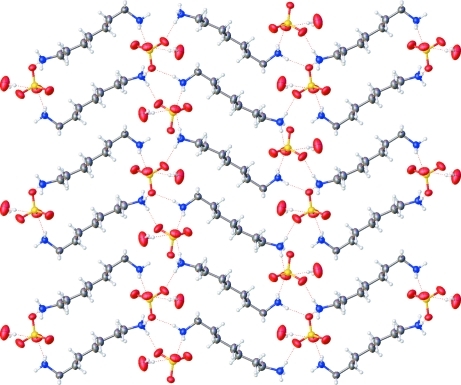
Packing arrangement of the title compound viewed down the a axis. Hydrogen bonds are indicated by red dashed lines.

**Fig. 3.**
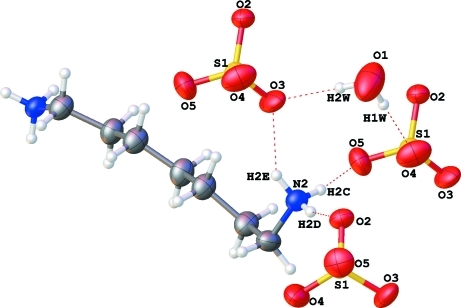
Close-up view of the title compound clearly showing selected hydrogen-bonding interactions. Hydrogen bonds are indicated by red dashed lines.

## supplementary crystallographic information

### Comment

The crystal structure of the title compound (I) adds to our current ongoing studies of long-chained diammonium inorganic mineral acid salts. Colourless crystals of heptane-1,7-diammonium sulfate hydrate were obtained from an attempt to synthesize heptane-1,7-diammonium sulfate. This material forms part of our structural chemistry study of the inorganic mineral acid salts of the *n*-alkyldiamines. A search of the Cambridge Structural Database (Version 5.32, Allen, 2002) revealed that this compound had not previously been determined.

The asymmetric unit of compound (I) contains one diammonium dication, one sulfate anion and one water molecule with all atoms occupying general positions. The hydrocarbon chain is also fully extended from N1 to C7 but then distorts from planarity through N2. This is evident in the C5—C6—C7—N2 torsion angle (-70.49°(18)). The molecular structure of (I) is shown in Fig. 1.

Fig. 2 illustrates the packing arrangement of the title compound (I). Single parallel-stacked layers of dications pack together with sulfate anions and water molecules inserted between the dication chains in line with the ammonium groups showing a distinct inorganic–organic layering effect that is a common feature of these long-chained diammonium salts. An extensive three-dimensional hydrogen-bonding network is formed.

A close-up view of selected hydrogen bonding interactions can be viewed in Fig. 3. The three-dimensional hydrogen bonding network is built and linked through hydrogen bonding interactions between the ammonium groups of the dication and the sulfate anions and water molecules. These extensive interactions are seen to contribute to the distortion from planarity of the ω-end of the diammonium cation. The hydrogen bond distances and angles for the title compound (I) can be found in Table 2.

### Experimental

Compound (I) was prepared by adding heptane-1,7-diamine (0.50 g, 3.84 mmol) to 33% sulfuric acid (H_2_SO_4_, 2 ml, 25.29 mmol, Merck) in a sample vial. The mixture was then refluxed at 363 K for 2 h. The solution was cooled at 2 K h^-1^ to room temperature. Colourless crystals of heptane-1,7-diammonium sulfate hydrate were collected and a suitable single-crystal was selected for the X-ray diffraction study.

### Refinement

H atoms were geometrically positioned and refined in the riding-model approximation, with C—H = 0.97 Å, N—H = 0.89 Å, and *U*_iso_(H) = 1.2*U*_eq_(C) or 1.5*U*_eq_(N). For (I), the highest peak in the final difference map is 0.87 Å from O4 and the deepest hole is 0.50 Å from S1.

### Crystal data

**Table:**

C_7_H_20_N_2_^2+^·SO_4_^2^^−^·H_2_O	*F*(000) = 536
*M**_r_* = 246.33	*D*_x_ = 1.266 Mg m^−^^3^
Monoclinic, *P*2_1_/*n*	Mo *K*α radiation, λ = 0.71073 Å
Hall symbol: -P 2yn	Cell parameters from 9981 reflections
*a* = 5.7527 (1) Å	θ = 2.2–28.2°
*b* = 22.3459 (4) Å	µ = 0.26 mm^−^^1^
*c* = 10.0908 (2) Å	*T* = 295 K
β = 95.180 (1)°	Block, colourless
*V* = 1291.87 (4) Å^3^	0.48 × 0.44 × 0.14 mm
*Z* = 4	

### Data collection

**Table:**

Bruker SMART CCD diffractometer	3220 independent reflections
Radiation source: fine-focus sealed tube	2626 reflections with *I* > 2σ(*I*)
graphite	*R*_int_ = 0.029
φ and ω scans	θ_max_ = 28.3°, θ_min_ = 1.8°
Absorption correction: multi-scan (*APEX2* AXScale; Bruker, 2008)	*h* = −7→7
*T*_min_ = 0.887, *T*_max_ = 0.965	*k* = −29→29
33427 measured reflections	*l* = −13→13

### Refinement

**Table:**

Refinement on *F*^2^	Primary atom site location: structure-invariant direct methods
Least-squares matrix: full	Secondary atom site location: difference Fourier map
*R*[*F*^2^ > 2σ(*F*^2^)] = 0.037	Hydrogen site location: inferred from neighbouring sites
*wR*(*F*^2^) = 0.106	H-atom parameters constrained
*S* = 1.03	*w* = 1/[σ^2^(*F*_o_^2^) + (0.0582*P*)^2^ + 0.3142*P*] where *P* = (*F*_o_^2^ + 2*F*_c_^2^)/3
3220 reflections	(Δ/σ)_max_ = 0.001
138 parameters	Δρ_max_ = 0.36 e Å^−^^3^
0 restraints	Δρ_min_ = −0.34 e Å^−^^3^

### Special details

**Table:**

Geometry. All e.s.d.'s (except the e.s.d. in the dihedral angle between two l.s. planes) are estimated using the full covariance matrix. The cell e.s.d.'s are taken into account individually in the estimation of e.s.d.'s in distances, angles and torsion angles; correlations between e.s.d.'s in cell parameters are only used when they are defined by crystal symmetry. An approximate (isotropic) treatment of cell e.s.d.'s is used for estimating e.s.d.'s involving l.s. planes.
Refinement. Refinement of *F*^2^ against ALL reflections. The weighted *R*-factor *wR* and goodness of fit *S* are based on *F*^2^, conventional *R*-factors *R* are based on *F*, with *F* set to zero for negative *F*^2^. The threshold expression of *F*^2^ > σ(*F*^2^) is used only for calculating *R*-factors(gt) *etc*. and is not relevant to the choice of reflections for refinement. *R*-factors based on *F*^2^ are statistically about twice as large as those based on *F*, and *R*- factors based on ALL data will be even larger.

### Fractional atomic coordinates and isotropic or equivalent isotropic displacement parameters (Å^2^)

**Table:**

	*x*	*y*	*z*	*U*_iso_*/*U*_eq_	
C1	0.1381 (3)	0.61468 (7)	0.89575 (17)	0.0465 (4)	
H1A	0.0280	0.6095	0.8180	0.056*	
H1B	0.0831	0.5915	0.9679	0.056*	
C2	0.3751 (3)	0.59183 (7)	0.86564 (17)	0.0452 (3)	
H2A	0.4775	0.5908	0.9474	0.054*	
H2B	0.4418	0.6194	0.8052	0.054*	
C3	0.3639 (3)	0.52966 (7)	0.80374 (18)	0.0485 (4)	
H3A	0.3229	0.5009	0.8697	0.058*	
H3B	0.2420	0.5289	0.7308	0.058*	
C4	0.5934 (3)	0.51120 (7)	0.75228 (18)	0.0491 (4)	
H4A	0.7111	0.5080	0.8271	0.059*	
H4B	0.6425	0.5424	0.6941	0.059*	
C5	0.5826 (3)	0.45218 (7)	0.67668 (17)	0.0459 (4)	
H5A	0.5538	0.4199	0.7375	0.055*	
H5B	0.4531	0.4534	0.6081	0.055*	
C6	0.8066 (3)	0.43935 (6)	0.61273 (17)	0.0459 (4)	
H6A	0.8377	0.4726	0.5552	0.055*	
H6B	0.9344	0.4372	0.6822	0.055*	
C7	0.8037 (3)	0.38213 (7)	0.53201 (15)	0.0447 (3)	
H7A	0.6643	0.3812	0.4703	0.054*	
H7B	0.9382	0.3813	0.4806	0.054*	
N1	0.1483 (2)	0.67881 (5)	0.93356 (12)	0.0375 (3)	
H1C	0.2474	0.6836	1.0057	0.056*	
H1D	0.0069	0.6911	0.9507	0.056*	
H1E	0.1965	0.7002	0.8669	0.056*	
N2	0.8078 (2)	0.32888 (5)	0.61913 (12)	0.0374 (3)	
H2C	0.9337	0.3302	0.6772	0.056*	
H2D	0.8121	0.2959	0.5699	0.056*	
H2E	0.6801	0.3284	0.6628	0.056*	
O1	0.7918 (3)	0.18441 (8)	0.8741 (2)	0.1013 (6)	
H1W	0.9211	0.2015	0.8472	0.152*	
H2W	0.7175	0.2197	0.8606	0.152*	
O2	0.29797 (18)	0.28426 (5)	1.00281 (10)	0.0408 (2)	
O3	0.54854 (19)	0.30097 (6)	0.82996 (12)	0.0561 (3)	
O4	0.2061 (2)	0.23929 (6)	0.78875 (13)	0.0647 (4)	
O5	0.1673 (2)	0.34542 (6)	0.81662 (12)	0.0595 (3)	
S1	0.30421 (5)	0.292599 (15)	0.85790 (3)	0.03274 (11)	

### Atomic displacement parameters (Å^2^)

**Table:**

	*U*^11^	*U*^22^	*U*^33^	*U*^12^	*U*^13^	*U*^23^
C1	0.0453 (8)	0.0395 (8)	0.0561 (9)	−0.0027 (6)	0.0128 (7)	−0.0056 (7)
C2	0.0444 (8)	0.0367 (8)	0.0553 (9)	0.0023 (6)	0.0080 (7)	−0.0067 (7)
C3	0.0528 (9)	0.0348 (7)	0.0592 (10)	−0.0008 (6)	0.0128 (7)	−0.0063 (7)
C4	0.0505 (9)	0.0341 (7)	0.0632 (10)	0.0009 (6)	0.0083 (7)	−0.0083 (7)
C5	0.0496 (8)	0.0318 (7)	0.0573 (9)	−0.0004 (6)	0.0112 (7)	−0.0050 (6)
C6	0.0507 (9)	0.0313 (7)	0.0571 (9)	−0.0015 (6)	0.0126 (7)	0.0019 (6)
C7	0.0583 (9)	0.0395 (8)	0.0376 (7)	0.0048 (7)	0.0106 (6)	0.0034 (6)
N1	0.0372 (6)	0.0388 (6)	0.0377 (6)	0.0044 (5)	0.0095 (5)	−0.0017 (5)
N2	0.0426 (6)	0.0321 (6)	0.0383 (6)	0.0009 (5)	0.0076 (5)	−0.0027 (5)
O1	0.0804 (11)	0.0641 (10)	0.1661 (19)	0.0094 (8)	0.0479 (12)	0.0226 (11)
O2	0.0442 (6)	0.0449 (6)	0.0343 (5)	0.0026 (4)	0.0096 (4)	0.0039 (4)
O3	0.0337 (6)	0.0865 (9)	0.0501 (7)	−0.0046 (5)	0.0140 (5)	0.0090 (6)
O4	0.0763 (9)	0.0612 (8)	0.0594 (7)	−0.0209 (6)	0.0219 (6)	−0.0252 (6)
O5	0.0583 (7)	0.0599 (7)	0.0583 (7)	0.0159 (6)	−0.0055 (6)	0.0138 (6)
S1	0.02925 (18)	0.03798 (19)	0.03160 (18)	−0.00106 (12)	0.00604 (12)	0.00133 (12)

### Geometric parameters (Å, °)

**Table:**

C1—N1	1.4829 (19)	C6—H6A	0.9700
C1—C2	1.512 (2)	C6—H6B	0.9700
C1—H1A	0.9700	C7—N2	1.4782 (18)
C1—H1B	0.9700	C7—H7A	0.9700
C2—C3	1.522 (2)	C7—H7B	0.9700
C2—H2A	0.9700	N1—H1C	0.8900
C2—H2B	0.9700	N1—H1D	0.8900
C3—C4	1.519 (2)	N1—H1E	0.8900
C3—H3A	0.9700	N2—H2C	0.8900
C3—H3B	0.9700	N2—H2D	0.8900
C4—C5	1.522 (2)	N2—H2E	0.8900
C4—H4A	0.9700	O1—H1W	0.9003
C4—H4B	0.9700	O1—H2W	0.9022
C5—C6	1.520 (2)	O2—S1	1.4777 (10)
C5—H5A	0.9700	O3—S1	1.4703 (11)
C5—H5B	0.9700	O4—S1	1.4674 (12)
C6—C7	1.515 (2)	O5—S1	1.4586 (12)
			
N1—C1—C2	111.28 (12)	C7—C6—H6A	108.6
N1—C1—H1A	109.4	C5—C6—H6A	108.6
C2—C1—H1A	109.4	C7—C6—H6B	108.6
N1—C1—H1B	109.4	C5—C6—H6B	108.6
C2—C1—H1B	109.4	H6A—C6—H6B	107.6
H1A—C1—H1B	108.0	N2—C7—C6	111.14 (12)
C1—C2—C3	112.65 (13)	N2—C7—H7A	109.4
C1—C2—H2A	109.1	C6—C7—H7A	109.4
C3—C2—H2A	109.1	N2—C7—H7B	109.4
C1—C2—H2B	109.1	C6—C7—H7B	109.4
C3—C2—H2B	109.1	H7A—C7—H7B	108.0
H2A—C2—H2B	107.8	C1—N1—H1C	109.5
C4—C3—C2	112.46 (13)	C1—N1—H1D	109.5
C4—C3—H3A	109.1	H1C—N1—H1D	109.5
C2—C3—H3A	109.1	C1—N1—H1E	109.5
C4—C3—H3B	109.1	H1C—N1—H1E	109.5
C2—C3—H3B	109.1	H1D—N1—H1E	109.5
H3A—C3—H3B	107.8	C7—N2—H2C	109.5
C3—C4—C5	114.14 (13)	C7—N2—H2D	109.5
C3—C4—H4A	108.7	H2C—N2—H2D	109.5
C5—C4—H4A	108.7	C7—N2—H2E	109.5
C3—C4—H4B	108.7	H2C—N2—H2E	109.5
C5—C4—H4B	108.7	H2D—N2—H2E	109.5
H4A—C4—H4B	107.6	H1W—O1—H2W	88.6
C6—C5—C4	112.23 (13)	O5—S1—O4	110.28 (8)
C6—C5—H5A	109.2	O5—S1—O3	110.06 (8)
C4—C5—H5A	109.2	O4—S1—O3	110.21 (8)
C6—C5—H5B	109.2	O5—S1—O2	108.90 (7)
C4—C5—H5B	109.2	O4—S1—O2	109.00 (7)
H5A—C5—H5B	107.9	O3—S1—O2	108.35 (6)
C7—C6—C5	114.75 (13)		
			
N1—C1—C2—C3	−170.09 (14)	C3—C4—C5—C6	172.97 (15)
C1—C2—C3—C4	170.28 (15)	C4—C5—C6—C7	−178.04 (14)
C2—C3—C4—C5	−173.90 (15)	C5—C6—C7—N2	−70.49 (18)

### Hydrogen-bond geometry (Å, °)

**Table:**

*D*—H···*A*	*D*—H	H···*A*	*D*···*A*	*D*—H···*A*
N1—H1C···O3^i^	0.89	1.97	2.8617 (17)	176
N1—H1D···O2^ii^	0.89	1.94	2.8250 (15)	177
N1—H1E···O4^iii^	0.89	1.92	2.8106 (17)	174
N2—H2C···O5^iv^	0.89	1.89	2.7643 (17)	168
N2—H2D···O2^v^	0.89	1.91	2.7859 (15)	166
N2—H2E···O3	0.89	2.01	2.7770 (16)	144
O1—H1W···O4^iv^	0.90	1.98	2.880 (2)	180
O1—H2W···O3	0.90	2.07	2.971 (2)	180

Symmetry codes: (i) −*x*+1, −*y*+1, −*z*+2; (ii) −*x*, −*y*+1, −*z*+2; (iii) −*x*+1/2, *y*+1/2, −*z*+3/2; (iv) *x*+1, *y*, *z*; (v) *x*+1/2, −*y*+1/2, *z*−1/2.
